# Study of hepatitis B and C prevalence and adherence to standards of biosecurity on manicures and/or pedicures in Brazil

**DOI:** 10.1186/1753-6561-5-S6-P220

**Published:** 2011-06-29

**Authors:** ACDSD Oliveira, AM Costa e Silva, S Scota, R Focaccia

**Affiliations:** 1 Educação continuada, Instituto de Infectologia Emilio Riba, São Paulo, Brazil

## Introduction / objectives

The habit of removing the nails cuticles of hands and feet is a typical cultural practice in Brazil and can be an important factor for hepatitis B and C infection. We conducted a seroepidemiological survey of hepatitis B and C in professional manicures/pedicures in salons in Sao Paulo - Brazil, with the aim of estimating the prevalence of serological markers of HBV and HCV infection on manicures/pedicures; get to know the information level of that have about transmission routes and Prevention of Hepatitis B and C, evaluate the perception degree of risk exposure accidental infectious agents and check the use of biosafety norms in the work routine of these professionals.

## Methods

This is a descriptive, cross sectional prospective study. The survey involved 100 participants manicures/pedicures from beauty salons, by random drawing. An individual for information about the characteristics participants; simultaneously questionare has been applyed was blood collected sample for the detection of serological markers of HBV and HCV of each participant.

## Results

Prevalence estimates were found in 8% of HBV and 2% of HCV. Membership biosafety standards for professionals were relatively low and inadequate. It was found that the degree of knowledge about routes of transmission, prevention, biosecurity standards and risk perception of infectious agents in their professional activity, was low. Manicures and pedicures are a group with increased risk factors, which determine a likely greater exposure to infection with viral hepatitis than the general population and all ways of prevention must be used to protect there health.

## Conclusion

It important to raise awareness manicures and pedicures becomes for the use of individual protection in their routine work to prevent future disease.

## Disclosure of interest

None declared.

